# MEP glia share molecular features with oligodendrocytes while maintaining a distinct hybrid signature

**DOI:** 10.17912/micropub.biology.001970

**Published:** 2026-01-21

**Authors:** Tessa C Dallo, Laura Fontenas

**Affiliations:** 1 Stiles-Nicholson Brain Institute Neuroscience Graduate Program, Florida Atlantic University, Boca Raton, Florida, United States; 2 Department of Biological Sciences, Florida Atlantic University, Boca Raton, Florida, United States

## Abstract

Motor Exit Point (MEP) glia are spinal cord-derived glial cells that myelinate peripheral motor axons, bridging the central and peripheral nervous systems. They have a hybrid profile, sharing features with oligodendrocytes and Schwann cells. Yet, significant gaps remain in our understanding of complex MEP glial lineage and identity. MEP glia express neural tube and canonical oligodendrocyte lineage markers *olig2* and *nkx2.2a* , as well as the neural crest marker *foxd3* . Here, we show that the oligodendrocyte markers *olig1* and *plp1b* are not expressed in MEP glia. These findings refine the molecular signature of MEP glia, enhancing their peripheral identity.

**Figure 1. MEP glia express some, but not all, CNS markers:**
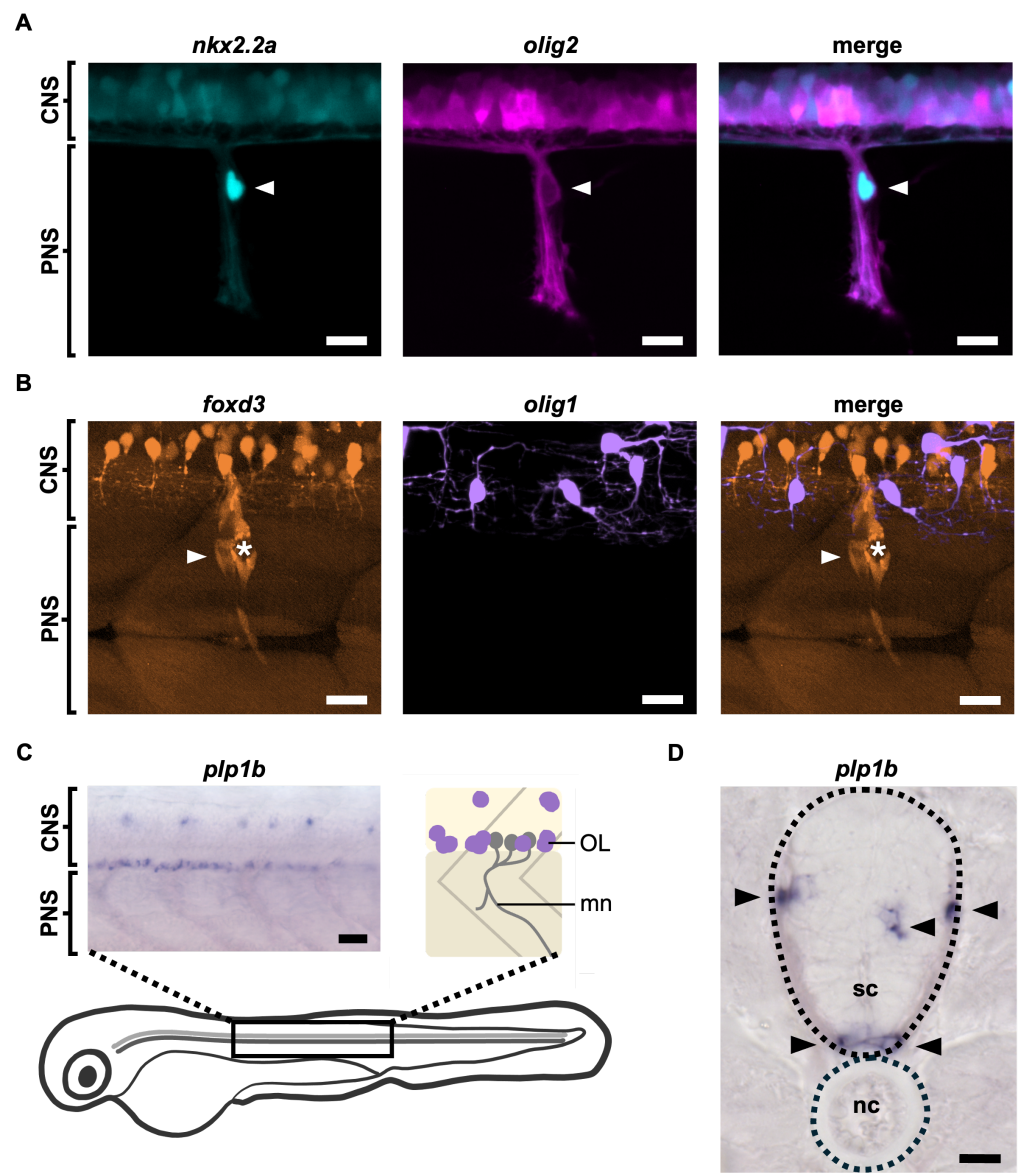
(A) Lateral views of a peripheral motor nerve in a *tg(nkx2.2a:nls:eGFP);tg(olig2:dsRed2)* zebrafish larva demonstrating *nkx2.2a* (cyan) and *olig2* (magenta) expression in MEP glia (white arrows) at 55 hpf. N = 60/60 nerves from 10 larvae. Scale bars: 10 µm. (B) Lateral views of a peripheral motor nerve in a *gt(foxd3:mCherry);tg(olig1:gal4;uas:eGFP)* zebrafish larva showing that MEP glia (white arrows) express *foxd3* (orange), but not *olig1* (purple) at 3 dpf. White asterisks denote the dorsal root ganglion. N = 60/60 nerves from 10 larvae. Scale bars: 15 µm. (C) Whole-mount lateral view and schematic of a *plp1b* RNA *in situ* hybridization in a 4 dpf zebrafish larva showing expression in oligodendrocytes in the spinal cord exclusively. N = 20/20 larvae. CNS: central nervous system; PNS: peripheral nervous system; OL: oligodendrocyte; mn: motor nerve. Scale bar: 25 µm. (D) Cross-section of a 4 dpf zebrafish larva *plp1b* *in situ* hybridization showing localization of *plp1b* RNA within spinal cord regions where oligodendrocytes are typically found (black arrows). No signal is detected at the MEP TZ outside of the CNS. N = 200/200 sections from 10 larvae. sc: spinal cord; nc: notochord. Scale bar: 15 µm.

## Description

Myelinating glia in the vertebrate nervous system are traditionally distinguished by their developmental origin and their function: oligodendrocytes originate from and myelinate axons in the central nervous system (CNS) (Almeida et al., 2011), and Schwann cells originate from and myelinate axons in the peripheral nervous system (PNS) (Jessen & Mirsky, 1999; Lyons et al., 2005). However, at transition zones (TZs) where central and peripheral nervous tissues meet, this dichotomy is not abrupt (Fontenas, 2023; Fontenas & Kucenas, 2018, 2021; Smith et al., 2014). In zebrafish, a distinct and dynamic population of glial cells, known as motor exit point (MEP) glia, can cross MEP TZs (Fontenas & Kucenas, 2018, 2021). Unlike oligodendrocytes and Schwann cells, which remain within their respective domains, MEP glia originate in the lateral floor plate of the neural tube and migrate out of the CNS to myelinate peripheral motor axons (Fontenas & Kucenas, 2018, 2021). They are a centrally derived population of peripheral myelinating glia that have a specialized role to maintain the CNS-PNS boundary, ensheathing the region between Schwann cells in the PNS and oligodendrocytes in the spinal cord (Fontenas & Kucenas, 2018).

In addition to their central origin and peripheral functioning, MEP glia have a hybrid identity, uniquely expressing both CNS-associated and PNS-associated markers (Fontenas & Kucenas, 2018, 2021). Like both oligodendrocytes and Schwann cells, they express *sox10* , a transcription factor essential for specifying and maintaining myelinating glial fates in both fish and mammalian systems (Britsch et al., 2001; Dutton et al., 2001; Fontenas & Kucenas, 2018, 2021; Smith et al., 2014; Stolt et al., 2002). Prior work has demonstrated that MEP glia also express oligodendrocyte lineage markers *olig2* (Fontenas & Kucenas, 2018, 2021) and *nkx2.2a* (Fontenas & Kucenas, 2021). Consistent with these findings, using *tg* ( *olig2:dsRed2)* ; *tg(nkx2.2a:nls-egfp)* double-transgenic larvae and *in vivo* confocal imaging, we observed expression of *olig2* and *nkx2.2a* in MEP glia at 55 hours post fertilization (hpf), when they populate peripheral motor nerves ( Fig. 1A ). Although *olig2* expression diminishes as MEP glia exit the spinal cord, a transient signal can still be detected (Fontenas & Kucenas, 2021). In contrast, *nkx2.2a* expression is robust and remains strong throughout post-embryonic stages (Fontenas & Kucenas, 2021). These markers are indicative of the origin of MEP glia from radial glia precursors, which are also known to generate motor neurons, oligodendrocytes, interneurons, and perineural glia (Fontenas & Kucenas, 2021).

Our previous studies show that MEP glia also express *foxd3* , a transcription factor associated with neural crest-derived glia (Fontenas & Kucenas, 2018, 2021; Smith et al., 2014). *Foxd3* is required for MEP glia to delaminate from the lateral floor plate and is also expressed in Schwann cells and dorsal root ganglia (Fontenas & Kucenas, 2021; Gilmour et al., 2002; Hochgreb-Hägele & Bronner, 2013). To further characterize MEP glia expression patterns, we imaged *tg(olig1:Gal4;UAS:egfp);gt(foxd3:mcherry)* larvae at 3 days post fertilization (dpf), a developmental timepoint that corresponds to oligodendrocyte *olig1* expression. Although MEP glia express several CNS-associated markers known as neural tube domain identity markers, they do not express *olig1* ( Fig. 1B ), a transcription factor restricted to oligodendrocyte lineage cells and that contributes to their proper differentiation and myelination (Dai et al., 2015; Li et al., 2007). This highlights another key molecular distinction between MEP glia and oligodendrocytes, suggesting that MEP glia only partially overlap with the oligodendrocyte differentiation program and adopt a more peripheral identity.

Because myelinating MEP glia express *myelin basic protein* ( *mbp* ), we next sought to investigate whether myelinating MEP glia express *proteolipid protein 1b* ( *plp1b* ), a major constituent of oligodendrocyte myelin sheaths (Brösamle & Halpern, 2002; Emery & Lu, 2015). Using an *in situ* hybridization detecting *plp1b* on 4 dpf larvae, we found that *plp1b* expression is limited to the CNS ( Fig. 1C ). To more precisely assess its spatial distribution, larvae were cryosectioned, and cross sections of the spinal cord were imaged. This revealed robust *plp1b* signal exclusively within the spinal cord, localized to the lateral, ventral, and medial regions occupied by myelinating oligodendrocytes ( Fig. 1D ). No *plp1b* signal was detected along peripheral motor nerves, where MEP glia myelinate axons. Other oligodendrocyte markers, such as *myelin regulatory factor* ( *myrf* ), are also absent, based on single-cell RNA sequencing data (Scott et al., 2021).

These findings refine the hybrid molecular identity of MEP glia, highlighting their divergence from an oligodendrocyte lineage trajectory. These data further establish MEP glia as a unique myelinating cell type at the CNS-PNS interface.

## Methods

Husbandry and generation of embryos: All animal studies were approved by the Florida Atlantic University (FAU) Animal Care and Use Committee. Zebrafish strains used in this study were: AB*, *Tg(nkx2.2a(3.5):nls-egfp) ^uva1^* (Fontenas & Kucenas, 2021), *Tg(olig2:dsRed2) ^vu19^* (Shin et al., 2003), *Tg(olig1:Gal4) ^fla1^* (this paper), *Tg(UAS:egpf) ^nkuasgfp1a^* (Asakawa et al., 2008), and *Gt(foxd3:mcherry) ^ct110R^* (Hochgreb-Hägele & Bronner, 2013). Embryos were raised at 28.5 °C in egg water and staged by hpf and dpf. Zebrafish cannot be sexed until adulthood; therefore, embryos of undetermined sex were used. Pigmentation was inhibited in embryos using phenyl-thiourea (PTU) (0.004%) in egg water. All studies were conducted using stable, germline transgenic lines.

Fish were housed and maintained in our fish facility at FAU under standard laboratory conditions. Adult zebrafish strains were kept at 28 °C on a 14-hour light, 10-hour dark cycle. To generate embryos, adult male and female zebrafish were housed in breeding tanks overnight and allowed to spawn naturally.

*In vivo* confocal imaging: Embryos were anesthetized with 0.01% 3-aminobenzoic acid ester (Syncaine) and embedded in 0.8% low-melting point agarose in a 35-mm glass-bottom imaging dish. Egg water containing PTU and Tricaine was added to the imaging dish after mounting. Images were captured using a Dragonfly inverted spinning disk confocal microscope with a motorized stage and a 40X/1.15 numerical aperture water immersion objective. Images were processed in Imaris to change channel colors, adjust levels and contrast, and crop the field of view.

*In situ* hybridization: Larvae were fixed at 4 dpf in 4% paraformaldehyde 1X phosphate-buffered saline at 4 °C overnight and stored in 100% methanol at -20 °C until processing. The *plp1b* RNA probe was generated using the following primers: plp1b FWD TCTCTGGAGTGAGCGAACGA and plp1b REV taatacgactcactatagCAGATCAGAGCGAGCACGTA; and t7 RNA polymerase. Whole-mount *in situ* hybridization was performed following standard protocols. Samples were imaged whole mount by embedding in 0.8% low-gelling temperature agarose in a glass-bottom 35 mm imaging dish. Samples were embedded in 1.5% agar/5% sucrose cryoblocks and sectioned using a Leica CM1950 cryostat. Brightfield whole-mount and sectioned images were obtained using a Keyence All-in-One BZ-X810 fluorescence microscope at 10X and 40X, respectively. Images were imported into Adobe Photoshop to adjust levels, contrast, and cropping.

## Reagents

**Table:** 

**REAGENT or RESOURCE**	**SOURCE**	**IDENTIFIER**
**Antibody**		
Sheep Anti-Digoxigenin-AP, Fab fragments	Sigma	Cat#11093274910; RRID: AB_514497
**Chemicals and Recombinant Proteins**		
3-Aminobenzoic acid ester (Syncaine)	Pentair	TRS1
1-Phenyl-2-thiourea (PTU)	Sigma	Cat#P7629; CAS number 103-85-5
Paraformaldehyde (PFA)	Sigma	Cat#158127
Agarose, low gelling temperature	Sigma	Cat#A94114
DIG RNA labeling mix	Sigma	Cat#11 277 073 910
**Enzyme**		
T7 RNA polymerase	New England Biolabs	Cat#M0251
**Experimental Models: Organisms/Strains**		
Zebrafish: AB*	ZIRC	&nbsp;
Zebrafish: *Tg(nkx2.2a(3.5):nls-eGFP) ^uva1^*	(Fontenas & Kucenas, 2021)	ZDB-ALT-211118-4
Zebrafish: *Tg(olig2:dsRed2) ^vu19^*	(Shin et al., 2003)	ZDB-ALT-080321-2
Zebrafish: *Tg(olig1:Gal4) ^fla1^*	This paper	N/A
Zebrafish: *Tg(5XUAS:eGPF) ^nkuasgfp1a^*	(Asakawa et al., 2008)	ZDB-ALT-080528-1
Zebrafish: *Gt(foxd3:mCherry) ^ct110R^*	(Hochgreb-Hägele & Bronner, 2013)	ZDB-ALT-130314-2
**Oligonucleotides**		
Plp1b FWD TCTCTGGAGTGAGCGAACGA	This paper	N/A
t7 + Plp1b REV taatacgactcactatag CAGATCAGAGCGAGCACGTA	This paper	N/A
**Software and Algorithms**		
Imaris	Oxford Instruments	Imaris
Adobe Photoshop 2025.23.0.0	Adobe	Adobe
